# Face identity and facial expression representations with adaptation paradigms: New directions for potential applications

**DOI:** 10.3389/fpsyg.2022.988497

**Published:** 2022-12-19

**Authors:** Kazusa Minemoto, Yoshiyuki Ueda

**Affiliations:** Institute for the Future of Human Society, Kyoto University, Kyoto, Japan

**Keywords:** face perception, face recognition, representation, aftereffect, norm-based coding, social message, development, autism spectrum disorder (ASD)

## Abstract

Adaptation and aftereffect are well-known procedures for exploring our neural representation of visual stimuli. It has been reported that they occur in face identity, facial expressions, and low-level visual features. This method has two primary advantages. One is to reveal the common or shared process of faces, that is, the overlapped or discrete representation of face identities or facial expressions. The other is to investigate the coding system or theory of face processing that underlies the ability to recognize faces. This study aims to organize recent research to guide the reader into the field of face adaptation and its aftereffect and to suggest possible future expansions in the use of this paradigm. To achieve this, we reviewed the behavioral short-term aftereffect studies on face identity (i.e., who it is) and facial expressions (i.e., what expressions such as happiness and anger are expressed), and summarized their findings about the neural representation of faces. First, we summarize the basic characteristics of face aftereffects compared to simple visual features to clarify that facial aftereffects occur at a different stage and are not inherited or combinations of low-level visual features. Next, we introduce the norm-based coding hypothesis, which is one of the theories used to represent face identity and facial expressions, and adaptation is a commonly used procedure to examine this. Subsequently, we reviewed studies that applied this paradigm to immature or impaired face recognition (i.e., children and individuals with autism spectrum disorder or prosopagnosia) and examined the relationships between their poor recognition performance and representations. Moreover, we reviewed studies dealing with the representation of non-presented faces and social signals conveyed *via* faces and discussed that the face adaptation paradigm is also appropriate for these types of examinations. Finally, we summarize the research conducted to date and propose a new direction for the face adaptation paradigm.

## 1 Introduction

The face is an important visual stimulus for our social life and it has attracted significant interest in the study of psychology. Various paradigms have been used to explore the perception and recognition of face identity and facial expressions. One useful tool is the adaptation and aftereffect method, also referred to as the “psychologist’s microelectrode” (Frisby, 1979). Perception of a given object stimulus (target, S2) can be biased or impaired by another stimulus, presented before (S1) the target. *Aftereffect* is a change in perception of a sensory stimulus due to the viewing of S1. At the behavioral level, this phenomenon is brought by *forward masking* (inhibition)/*priming* (facilitation)/*adaptation* (biased perception) (Walther et al., 2013; Mueller et al., 2020 for a review). At the neural level, it is usually observed as signal (activation) reduction for repeated stimulus presentation. Moreover, a very extreme case of adaptation is the so-called repetition suppression, when the same stimulus is presented several times in a row (e.g., Grill-Spector et al., 2006; Schweinberger and Neumann, 2016 for a review). The typical adaptation experimental procedure called S1 as *adaptor* or *adaptation stimuli*, and S2 as *test stimuli*. In this procedure, participants were asked to keep looking at the adaptation stimulus, and to respond to the test stimuli with answers such as who they looked like in the pre-learned individual or which facial expression they expressed. For example, after viewing the adaption stimulus of an individual (e.g., Jim) or a facial expression (e.g., happy), the perception of participant is changed, resulting that they cannot recognize the test stimuli as Jim or happy expressions though they recognize the same stimuli as Jim or happy expressions before the adaptation.

Previously, the adaptation paradigm was used in low-level (or simple feature) perception of visual stimuli, such as perception of color (Webster, 1996) and tilt (Gibson and Radner, 1937); it was later applied to higher-level (or visually more complex stimulus) cognition concerning face perception: configuration of a face (Webster and MacLeod, 2011 for a review), face identity (e.g., Leopold et al., 2001; Rhodes and Jeffery, 2006), facial expression (e.g., Hsu and Young, 2004; Webster et al., 2004), gaze direction (e.g., Jenkins et al., 2006; Seyama and Nagayama, 2006; Clifford and Palmer, 2018 for a review), masculinity/femininity (e.g., Webster et al., 2004; Little et al., 2005; Jaquet and Rhodes, 2008), ethnicity (e.g., Webster et al., 2004), and attractiveness (e.g., Rhodes et al., 2003). Two major problems that must be solved using this method are discussed. One problem is to reveal the common (or shared) process of facial information. If two facial information are processed in the same process, based on the same neural representation, the prior presentation of the face could have an aftereffect on the recognition of the subsequent face. The second is to reveal the neural representations of the face. The latter issue has been examined under the hypothesis of norm-based coding proposed by Valentine (1991), in which faces are assumed to be represented in a mental space, centered on the average of all faces that each person has seen. Each face was identified based on its distance and direction from the center point in this space. According to this, adapting to a certain face recalibrates and temporally shifts the center point to the adaptation stimuli in the space, resulting in a change in subsequent perception (see the detailed discussion in section 3.1).

This study first overview what face adaptation studies (mainly using behavioral paradigms) revealed regarding the representation of face identity (i.e., who it is) and facial expression (e.g., happiness, anger). Then, beyond the scope of previous studies, we introduce the recent findings that can be revealed using the face adaptation paradigm, and discuss new potential applications of it. Specifically, in section 2, we summarize the basic procedures of facial adaptation and its aftereffects. Considering previous eminent reviews of face adaptation (Webster and MacLeod, 2011; Rhodes and Leopold, 2012; Strobach and Carbon, 2013; Mueller et al., 2020), recent findings and other information not presented therein have also been introduced. Some terms are used ambiguously, and we have noted this and redefined them. This section would help readers who have set foot in this field. In section 3, we review studies that have investigated facial representations of individual identity and facial expression, as well as studies that have expanded the scope of applicability of the facial adaptation paradigm. Using the face adaptation paradigms, some cognitive models concerning facial representation have been examined. We discuss what has revealed by face adaptation studies the face and facial expression recognition models (Ekman and Friesen, 1971; Bruce and Young, 1986; Valentine, 1991; Haxby et al., 2000) and what remains unclear yet. Furthermore, studies investigating children and participants with atypical face recognition (i.e., autism and prosopagnosia) are mentioned. Participants with atypical face recognition, in particular, have not been well-mentioned in the existing review articles. In section 4, we further expand the scope of the face adaptation paradigm and suggest that it is also useful for examining representations of non-presented faces (i.e., the mental images and ensemble average of faces) and social signals conveyed through faces. This section is a review of new findings in recent years, showing that the face adaptation paradigm can shed light on what is still unclear in representations related to face. In section 5, we discuss the representation of faces in the current stage and suggest the future applicability of the facial adaptation paradigm.

## 2 Basic characteristics of face aftereffects

First, we summarize the basic characteristics of face aftereffects to clarify the effectiveness of the adaptation method. In particular, aftereffects have been investigated in low-level visual features, and many studies have explored the relationship between stimuli and whether the two share common mechanisms.

### 2.1 Adaptation for high- and low-level visual processing

The face includes many low-level visual features (e.g., color, tilt, or shape). In the early face and facial expression adaptation research, there was a great focus on whether the face aftereffect was due to high-level visual processing specific to the face or the results of retinotopy or inheritance from aftereffects of low-level visual processing. One of the popular procedures to examine this is to change the positions or physical sizes of the adaptation and test stimuli. Previous research has reported that face aftereffects persist even when the aftereffects of low-level visual features collapse (Leopold et al., 2001; Rhodes et al., 2007 in face identity; Hsu and Young, 2004; Burton et al., 2016; Zamuner et al., 2017 in facial expressions).

In addition, studies using composite and hybrid faces have demonstrated that face adaptation differs from adaptation in low-level visual processing (Butler et al., 2008; Laurence and Hole, 2012). Face and facial expressions are related to both featural processing, such as eye and mouth, and configural (holistic) processing, such as the spatial arrangement of facial parts. Composite and hybrid faces were used to examine the effect of configural processing on the recognition of faces and facial expressions. A composite face is a photograph in which the top and bottom halves of a face are misaligned, and a hybrid face is a photograph of two different identities or expressions combined into one face (e.g., the top half expresses happiness and the bottom half expresses sadness). It is difficult to correctly recognize identity and facial expressions in these stimuli, although they have the same featural component as normal faces (Young et al., 1987; Calder et al., 2000). If the face aftereffect is based on low-level visual features, adaptation to a composite or hybrid face is expected to produce the same magnitude of aftereffects as does adaptation to normal faces. Butler et al. (2008) reported that a significant aftereffect was observed when participants adapted to normal and hybrid faces made of different images from the same facial expressions. However, an aftereffect was not observed when they adapted to hybrid faces made of different facial expressions. Moreover, Laurence and Hole (2012) reported that the composite faces in which participants could recognize the identity showed an aftereffect, while those in which participants could not recognize the identity did not show the aftereffect. These studies suggest that recognizability is an important factor in the face aftereffects.

However, the contribution of the aftereffect of low-level visual features cannot be denied as many studies have demonstrated it. The face aftereffect remains when the size and position of adaptation and test stimuli change, but it has been shown that the further away facial aftereffects are from the adaptation location, the weaker they become (Afraz and Cavanagh, 2008). In addition, only simple concave or convex curved lines and an isolated mouth from the real face are sufficient to cause the aftereffect of facial expressions, but these effects disappear when the presented position of adaptation and test are different (Xu et al., 2008). In addition, when the orientation of adaptation and test stimuli changed (the orientation of the adaptation and test stimuli rotated by 90^°^), the aftereffect of the expressions decreased (Swe et al., 2019). Note that the aftereffect remained when the test stimuli were rotated by 90^°^, suggesting that the orientation aftereffect could not fully explain the face aftereffect.

### 2.2 Temporal dynamics of face adaptation

After finding the face aftereffect, researchers were interested in its characteristics and found a commonality between the face aftereffect and low-level visual feature aftereffect.

With regard to temporal dynamics, there is a close relationship between the amount of face aftereffect and the presentation time: the increasing duration of the exposure to the adaptation stimuli builds up the aftereffect logarithmically, and the increasing duration of exposure to the test stimuli decays the aftereffect exponentially (Leopold et al., 2005; Rhodes et al., 2007; Burton et al., 2016). These effects were observed within a shorter adaptation period (e.g., 1 s). These dynamics occur in both face identity and facial expression aftereffects (Leopold et al., 2005; Rhodes et al., 2007; Burton et al., 2016). In addition, the time duration between adaptation and test stimuli (i.e., inter-stimulus intervals) also affects the size of the face aftereffect; that is, longer gaps lead to weaker aftereffects (Burton et al., 2016).

Temporal dynamics of face aftereffects suggest an answer to a question: As the faces are social stimuli, when the two faces are presented in succession, some meaning or context will alter the response to the test stimuli. If this is true, it would be expected that their responses would not be affected by the duration of either the presentation or gap durations between adaptation and test stimuli; however, the results showed their influence (Leopold et al., 2005; Rhodes et al., 2007; Burton et al., 2016). In summary, the change in response after prolonged viewing of the face is not the result of context or strategy due to the sequential presentation of the two faces, but is the result of the aftereffect.

Hsu and Young (2004) showed a facial expression aftereffect when the adaptation duration was 5,000 ms but not 500 ms, suggesting that a certain duration is necessary to produce the adaptation effect. However, it could not determine that 500 ms is the minimum length of exposure to elicit an aftereffect because it may change not only by adaptation duration but also by various factors, such as valence or its intensity of facial expressions or repetition of the adaptation stimulus. For example, aftereffects were observed by adaptation to angry expressions for 17 ms and happy expressions for 50 ms (Sou and Xu, 2019), and repeated presentation of a 500 ms adaptation face (Moriya et al., 2013). In addition, some neurophysiological studies have robustly reported the short-term adaptations (around 200 or 300 ms) reduce the face-sensitive neural activations of M170 in magneto-encephalographic (MEG) studies (Harris and Nakayama, 2007, 2008), N170 in event-related potentials (ERP) studies (Eimer et al., 2010; Nemrodov and Itier, 2011), and functional magnetic resonance imaging (fMRI) signal in fusiform cortex and posterior superior temporal sulcus (Winston et al., 2004).

Moreover, there are interaction between adaptation duration and position consistency of adaptation and test stimuli (Zimmer and Kovács, 2011 for a review). Kovács et al., 2005, 2007, 2008 investigated the N170 amplitude under conditions where the adaptation and test stimuli were presented at the same or different positions using an adaptation task of face gender. Results showed that the long-term (5,000 ms) adaptation duration induced the greater reduction in N170 amplitude when the adaptation and test stimuli were presented at the same location compared to the different locations, though no differences by location were observed for short-term (500 ms) adaptation duration. It suggests that the long-term face adaptation is position-specific, while the short-term face adaptation is position-invariant. The same results were reported in fMRI study and indicated that the activations of the right occipital face area reduced when the positions of the adaptation and test stimuli were the same after only long-term (4,500 ms) adaptation, but no differences were observed either when the positions of two stimuli were different or when adaptation duration was short (500 ms) (Kovács et al., 2008). On the other hand, the activations of the right fusiform face area reduced regardless the adaptation duration and the locations of two stimuli. These studies suggest that different adaptation durations are associated with different neural mechanisms.

## 3 What is revealed by face aftereffect?

Using the face adaptation “paradigms, some cognitive models concerning facial representation have been proposed. Here we introduce the famous face and facial expression models (Ekman and Friesen, 1971; Bruce and Young, 1986; Valentine, 1991; Haxby et al., 2000) and discuss what face aftereffect examined about them and what remains unclear yet.

### 3.1 Face representation

#### 3.1.1 Face identity

Early experiments on face aftereffect were conducted using distorted images of the face created by a circular Gaussian envelope so that the face elements were expanding or contracting relative to a midpoint on the nose (Webster and MacLin, 1999). It was found that the face appeared biased toward the opposite direction of the preceding presented stimulus. For example, the original face appears to expand after adapting to contracting faces. In addition, this face aftereffect was beyond the mere distortion of visual objects because the face aftereffect decreased when the orientations of the adaptation and test faces were different (i.e., upright vs. inverted). Adaptation to the original (non-distorted) image did not change the face perception. These results suggest that our perception of the face was normalized by what we saw immediately before.

**FIGURE 1:**
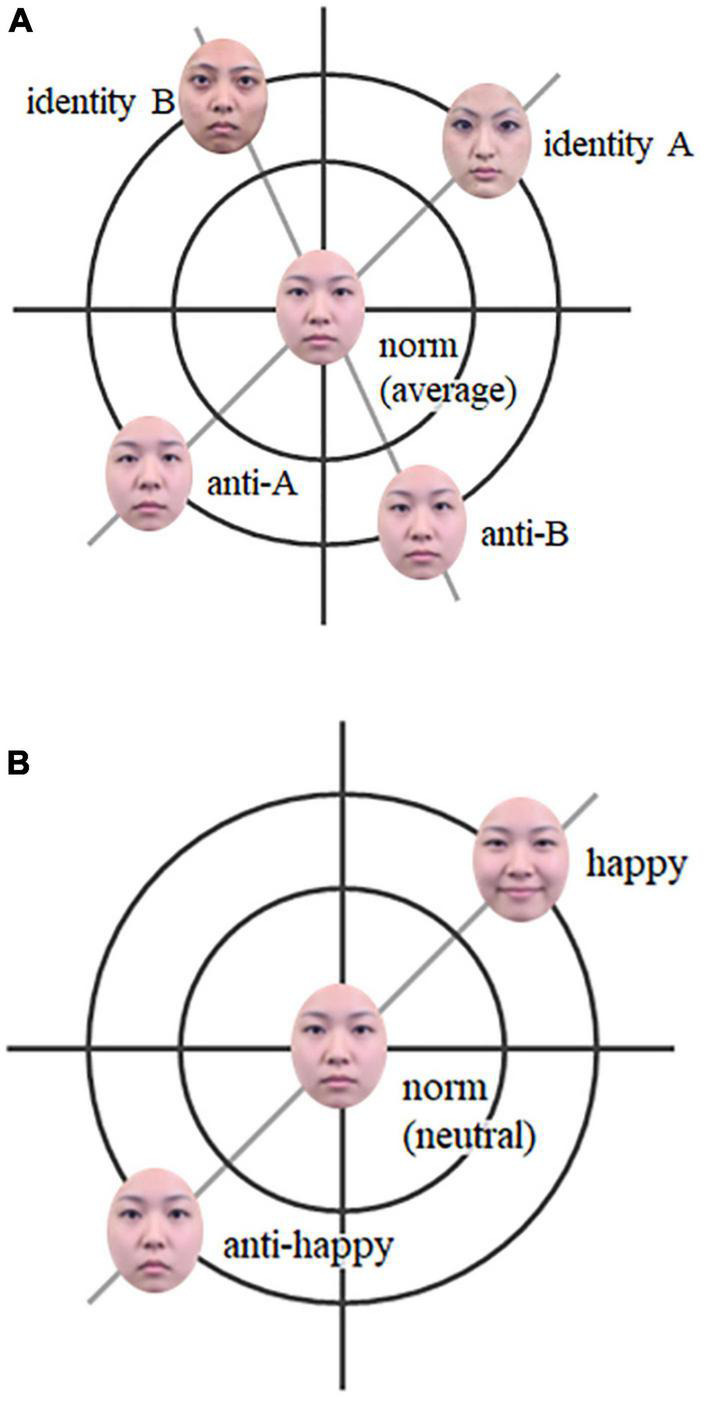
Schemas of the face-space. **(A)** Identity: two identities (identity A and B), their anti-faces (anti-A and anti-B), and norm (average). **(B)** Facial expression: a facial expression (happy), its anti-expression (anti-happy), and norm (neutral). The anti-faces were made from each identity or expression and norm (average or neutral) using morphing techniques.

Subsequently, adaptation research has examined an important face recognition framework called “face space” (Valentine, 1991) by using anti-face images (Leopold et al., 2001; Rhodes and Leopold, 2012 for a review). Face space is considered a multidimensional mental space centered on the norm face, and each face is represented in this space (see Figure 1). In accordance with this idea, people represent each face identity to refer to the distance from the center of mental space (it is called “norm”^1^), which was investigated using anti-faces (e.g., Leopold et al., 2001; Rhodes and Jeffery, 2006). *Anti-face*s were generated by making a face with features that were the physically opposite of the original face to the average face of multiple faces using *morphing* techniques (here, the average of multiple faces can be regarded as the substitution of the norm). For example, identity A have smaller eyes than the average face while anti-face of A (described “anti-A”) have bigger eyes than the average. Although the anti-face does not look like the original person from whom it was created, it lies on the same axis connecting the original face to the average face on the opposite side of the average face from the original face. This feature dimension through the original face, average (norm) face, and anti-face is referred to as the *identity trajectory* (Leopold et al., 2001). Leopold et al. (2001) showed that after adapting to the anti-face for 5 s, the intensity of the features needed to identify each person (i.e., stimulus identity thresholds) decreased, and the average face was frequently identified as the original person of the anti-face. These aftereffects seem to be the result of the temporal shift of the central point of the face space toward an adapting face to fit the neural populations, which have a limited response range, depending on the current situation^2^

Furthermore, Rhodes and Jeffery (2006) examined the differences between the aftereffects of two types of anti-faces. One is the *matching* anti-face, which lies on the same trajectory as the test face, and the other is the *mismatching* anti-face, which does not lie on the same trajectory as the test face but on the trajectory of another face. The latter was equally perceptually dissimilar to the original face, but with a different identity trajectory^3^. The results showed that adaptation to the matching anti-face had a larger aftereffect on the recognition of the target face compared to adaptation to the mismatching anti-face. Thus, the aftereffect of the face is a selective bias of the central point toward the adapting face in the face space, but not the simple contrast effect between two faces (see Figure 1; Rhodes and Jeffery, 2006).

In summary, the results of the identity aftereffect suggest that perception of faces could be based on the face space in which people refer to the direction and distance between the norm and the individual face. In addition, this face space is temporally and immediately recalibrated with what we see, even for seconds, and it is quite likely that this flexible updating occurs in real life. Accordingly, the aftereffect is not limited to the picture and occurs even when adapting to the video clip of a face (Petrovski et al., 2018).

#### 3.1.2 Facial expressions

Following the studies of the identity aftereffect, adaptation studies have focused on facial expressions to reveal their representation, in which two theories have long been the center of controversy. One is the categorical theory, which states that facial expressions are represented as discrete qualitative categories. The idea is led by Ekman and Friesen (1971) and assumes that there are six basic categories of facial expressions (anger, fear, disgust, happy, sad, and surprise) that are innate and universal and are often used in studies of facial expressions. The other is the continuous theory that argues facial expressions are represented in the circumplex model (Russell, 1980; Russell and Bullock, 1985). The main dimensions of this model are valence (unpleasant-pleasant) and arousal (high arousal-low arousal or sleepiness). In the latter theory, the boundaries of the categories are ambiguous and the category can change depending on the context in which the expression is presented (Carroll and Russell, 1996).

To investigate which of the two hypotheses is appropriate, the literature used the same and different categories of expressions as the adaptation and test stimuli. For the aftereffect between the same category of adaptation and test facial expressions, there is growing consensus that prolonged presentation of facial expressions leads to strong selective aftereffect (Hsu and Young, 2004; Webster et al., 2004; Juricevic and Webster, 2012), in which after adapting to happy facial expressions, the more intensely happy faces are needed to perceive happiness compared to before the adaptation (Hsu and Young, 2004; Webster et al., 2004; Juricevic and Webster, 2012). Likewise, after adapting to anti-happy facial expressions, participants can perceive happiness, even for less happy expressions (Skinner and Benton, 2010; Juricevic and Webster, 2012). On the other hand, for the aftereffect between the different categories of adaptation and test stimuli, some studies performed it but did not reach a consensus on their results. In Experiment 1 of Hsu and Young (2004), the facilitation aftereffect on the recognition of sad facial expressions (i.e., participants can perceive sadness even for less sad expressions) after adapting to happy facial expressions was presented, but this aftereffect was not found in Experiment 2, which employed the same procedure but a different identity face set. Juricevic and Webster (2012) found that adaptation to any of the six basic emotion categories did not facilitate the recognition of any category. The results showed that robust aftereffects were observed only in combinations of the same facial expressions, indicating that people could process facial expressions categorically. However, the aftereffect was observed in some combinations of different facial expressions, suggesting that the categories of facial expressions are not completely independent. Considering these studies, it is suggested that the categories of facial expressions are semantically independent, but their neural representations somewhat overlap.

Although the adaptation paradigm was expected to shed light on the discrete process, it was difficult to make a clear consensus between the two hypotheses because the aftereffects were found in both the same and different categories between the adaptation and test facial expressions (Hsu and Young, 2004; Rutherford et al., 2008; Pell and Richards, 2011; Juricevic and Webster, 2012). Therefore, the aim of this study shifted from showing which of the two hypotheses is correct to examining the representation of facial expressions, incorporating the concept of face space.

To elaborate on the representation of facial expressions, Cook et al. (2011) conducted an experiment based on the idea of Rhodes and Jeffery (2006), as mentioned in subsection 3.1.1. They used the anti-facial expression and the original facial expression as a pair (A and B) and the orthogonal faces pair (C and D), whose features are in the orthogonal direction against an A and B pair in the facial expression space. The results showed that selectivity aftereffects were observed when the adaptation stimulus was congruent with the test stimulus (e.g., the adaptation stimulus was either A or B, and the test stimulus was one of AB continua). When incongruent pairs were presented as the adaptation and test stimuli (e.g., the adaptation stimulus was either A or B, and the test stimulus was one of CD continua), no or fewer aftereffects were observed. These results suggest that facial expressions are represented by multidimensional facial spaces.

In addition, it has been reported that adaptation stimuli causing stronger aftereffects are associated with more intense facial expressions (Skinner and Benton, 2010; Burton et al., 2015; Rhodes et al., 2017; Hong and Yoon, 2018). These results suggest that we do not perceive facial expressions regardless of their intensity (if so, then an adaptation to a facial expression should always lead to a constant intense aftereffect), but that the perceptual bias due to adaptation shifts gradually as a function of the intensity of the adaptation stimuli. These results also support the norm-based coding of facial expressions.

#### 3.1.3 Relationship between identity and facial expression

As we can recognize facial expressions even if we do not know who the person is, earlier face models suggest that face identity and facial expression are processed distinctively (Bruce and Young, 1986; Haxby et al., 2000). However, some recent studies have argued that they are not independent of each other (Kaufmann and Schweinberger, 2004; Winston et al., 2004; Calder and Young, 2005). The adaptation paradigm is a good way to examine this using the same and different models of the same facial expression. Simultaneously, it is also possible to examine whether face identity and facial expressions can be mapped onto the same mental space. To investigate these questions, Fox and Barton (2007) compared the aftereffect with four combinations of adaptation and test stimuli: two types were from the same model with the same and different images, and the other two types were from different models with the same or different genders displaying facial expressions. The results showed that all combinations led to the same direction of the aftereffect, but the size of the aftereffect was smaller in the different model conditions than in the same model with the same image conditions. To determine whether differences in the aftereffect size were due to differences in image or model identity, they further compared conditions in which the same and different images of the same model showed comparable aftereffects across these conditions. The same patterns have been observed across different emotional expressions (Campbell and Burke, 2009; Pell and Richards, 2013). Ellamil et al. (2008) examined this aftereffect using faces that had the same shape (featural patterns) but were wrapped with the surfaces of different models. Although the featural pattern was the same, the size of the aftereffect decreased when the wrapped model differed from the adaptation model. These results suggest that there are identity-independent and identity-dependent representations of facial expressions. In addition, Song et al. (2015) reported that when adaptation and test stimuli had the same identity and expression configurations, the aftereffect was larger than when adaptation and test stimuli had the same identity but different expression configurations. By contrast, when their identities were different, the aftereffect was comparable, regardless of the expression configuration. This indicates that sensitivities to expression configurations were different between the identity-dependent and independent representations.

Furthermore, a positive correlation was observed between face identity aftereffect and facial expression aftereffect, while these aftereffects did not correlate with other features of the face (gaze direction) and the aftereffect using orientation stimuli (i.e., not face, but a geometric feature) (Rhodes et al., 2015). Their results suggest that there is a common representation of identity and expression. However, Rhodes et al. (2015) reported that the recognition ability of identity and expressions were predicted by identity aftereffects regardless of expression and expression aftereffects regardless of identity, suggesting identity-selective or expression-selective dimensions.

In summary, most studies have agreed that there are two components of face adaptation: one is the identity-independent representation, which results in the aftereffect regardless of model, and the other is the identity-dependent representation, which results in a larger aftereffect when adapting to the same model rather than a different model. These two components are represented as a multi-dimensional space because anti-expressions led to the aftereffect of whether the models in the adaptation and test stimuli were the same or different, and the weaker adaptation stimuli led to smaller aftereffects (Skinner and Benton, 2012). Interestingly, asymmetric results were observed in an identity recognition study; Fox et al. (2008) demonstrated that the identity aftereffect did not change depending on whether the conditions of adaptation and test stimuli were the same or different facial expressions. This suggests that identity can be independently represented by a facial expression component. As few studies have investigated this asymmetric effect, it is important to modify and revise the model of face recognition in the future.

### 3.2 Development and impairment investigated using face adaptation

People are experts at recognizing and discriminating between face identity and facial expressions. The norm-based representation supports this skill, as reviewed in the previous section, and some research has reported that there is a correlation between the recognition ability and the size of the aftereffect (Rhodes et al., 2014b,^2015^; Palermo et al., 2018). In this section, we review this face representation in people who are not as proficient as healthy adults, such as children or people who have impaired face recognition function. As developmental research on identity aftereffects was reviewed by Jeffery and Rhodes (2011), we summarize it simply and focus on the facial expressions and interaction of face identity and facial expressions in this section.

#### 3.2.1 Development of norm-based recognition

Face-recognition ability develops according to age (Vicari et al., 2000; Mondloch et al., 2003). An adaptation study has explored the developmental improvement in norm-based coding, and most studies have shown the same patterns of aftereffect in children aged 4–10 years as well as adults (Jeffery and Rhodes, 2011 for a review). Further, matching anti-face leads to larger aftereffects than mismatching anti-face even when matching anti-face and mismatching anti-face had the same perceptual dissimilarity, and the more intense adaptation stimuli led to larger aftereffects, suggesting that children over 4 years old may have multidimensional face space (Nishimura et al., 2008 with 8 years of age; Jeffery et al., 2010 with 4–6 years of age; Jeffery et al., 2011 with 5–9 years of age; Jeffery et al., 2013 with 4 years of age).

To our knowledge, only two studies have examined the development of aftereffects of facial expressions (Vida and Mondloch, 2009; Burton et al., 2013). Vida and Mondloch (2009) reported that the aftereffects of facial expressions were observed in the children’s group, but this depended on the pairs’ expressions (i.e., happy/sad or fear/anger). While children aged 5 years did not show adult-like aftereffects in happy/sad pairs, children aged 7 years showed adult-like aftereffects in these pairs. However, children aged 7 years did not show adult-like aftereffects in fear/angry pairs, whereas children aged 9 years showed adult-like aftereffects in these pairs. Considering that a previous study indicated that the development of sensitivity to fear and anger was slower than happiness and sadness (Vicari et al., 2000), the results suggest that the aftereffect of facial expression might depend on the developmental sensitivity of facial expressions. Burton et al. (2013) investigated whether the representation of facial expressions in 9 years-old children is norm-based or not, using anti-facial expressions with two types of strength. They found that the aftereffect occurred after adaptation to anti-facial expressions, and stronger adaptation led to a larger impact of aftereffects. These results supported the idea that children of these ages could represent facial expressions in multidimensional facial space as well as adults.

Studies with adults have suggested that there are identity-dependent and identity-independent components for the representation of facial expressions, while identity representation is independent of facial expressions (Fox and Barton, 2007; Fox et al., 2008; Skinner and Benton, 2012). Likewise, children may have the same two types of components because adaptation to the same identity as the test stimuli resulted in a larger aftereffect rather than adaptation to a different identity from the test stimuli (Vida and Mondloch, 2009). In addition, the identity aftereffect was not affected by whether the adaptation and test stimuli had the same or different facial expressions as 8 years-old children (Mian and Mondloch, 2012), showing an asymmetric representation between identity and facial expression.

Interestingly, an adult-like identity aftereffect was observed at age 4 years (Jeffery et al., 2013), but an adult-like facial expression aftereffect was observed over 7 years of age (Vida and Mondloch, 2009). However, it is premature to discuss this because there were different points in the stimuli used in these studies, such as whether they changed the model or if they used an anti-face. To achieve a deeper understanding of the development of facial expression and identity and to consider their relationships with each other, more research on the aftereffects of facial expressions is needed, such as experiments conducted under the same procedure for identity and facial expression, younger participants, or other expression categories.

#### 3.2.2 Impairment face recognition and aftereffect

Many types of atypical social communication have been reported, among which autism spectrum disorder (ASD) is a major cause of deficits in facial recognition (Harms et al., 2010; Weigelt et al., 2012). Adaptation paradigms have been used to examine norm-based representations. For face identity, studies have shown that adapting to the anti-face leads to an aftereffect in both autism and typical development (TD) groups (Pellicano et al., 2007; Rhodes et al., 2014a; Walsh et al., 2015), and it becomes stronger as the intensity (i.e., the distance from the norm) of the face increases (Rhodes et al., 2014a; Walsh et al., 2015). This suggests that a norm-based facial recognition system can be implemented for people with ASD. Moreover, studies have also shown that the aftereffect size was smaller in children with ASD than in those with TD (Pellicano et al., 2007 on ages 8–13; Rhodes et al., 2014a on ages 9–14). This effect was comparable between healthy adults and adults with ASD (Cook et al., 2014; Walsh et al., 2015). In summary, these results indicate that there are no large qualitative differences in norm-based identity representation between adults with TD and ASD, suggesting that the deficits in identity recognition in adults with ASD are not due to perceptual representations. However, there are differences between children with TD and ASD, suggesting that children with ASD are slower to become proficient in norm-based representations than those with TD.

Likewise, for facial expressions, adaptation to facial expressions led to an aftereffect, in which recognition of the original facial expressions could be facilitated, even in people with ASD (Rhodes et al., 2018; Hudac et al., 2021). The stronger intensity of adaptation stimuli also led to a larger aftereffect (Rhodes et al., 2018), suggesting that ASD could represent facial expressions in a norm-based manner. In addition, a smaller aftereffect size was found in children with ASD (Rhodes et al., 2018), although no difference was found in adults (Rutherford et al., 2012; Cook et al., 2014). Rutherford et al. (2012) reported that the response patterns after facial expression adaptation differed between individuals with ASD and TD. After adapting to negative emotions (e.g., fear), ASD participants tended to choose a sad label for the test stimulus of neutral faces, although TD participants tended to choose a happy label for them. These results suggest that participants with ASD encode facial expressions in a different mental facial expression-space than those with TD, in which negative and positive expressions are not opposites on the same axis. As there is some debate on the relationship between the categories of facial expressions, this point needs to be further investigated in the future.

Prosopagnosia is another well-known neuropsychological disorder that causes deficits in face recognition but has normal intelligence, memory, and low-level vision. The adaptation paradigm has also been used to reveal face coding systems and representations in this group. It is known that there are different types of prosopagnosia depending on the different causes of symptoms and impaired cognitive processes. The former is the *congenital prosopagnosia* (CP, also known as developmental prosopagnosia), who had no known brain injury but had difficulty recognizing face by nature and the *acquired prosopagnosia* (AP), who impaired their ability of face recognition due to acquired brain damage. The latter is the *apperceptive* and the *associative prosopagnosia*, which are dysfunctions of face recognition processes (De Renzi et al., 1991). It is suggested that each of those types is associated with a different cognitive stage of Bruce and Young (1986)’s model, which describes from face perception to name identification and separates the distinctive face cognitive processes into multiple stages (Corrow et al., 2016 for a review). The *apperceptive prosopagnosia* is impaired the structural encoding, which is the first stage in Bruce and Young’s model, resulting the failure of face perception. On the other hand, the *associative prosopagnosia* is impaired the face recognition units, the second stage in their model, resulting the impaired sense of familiarity and recall to the familiar faces though they can accurately perceive the facial structure.

Most adaptation studies for the prosopagnosia had been conducted for CP mainly. The pattern of identity aftereffects of CP was the same as that of the control participants, that is, adapting to anti-face enhanced identification of the original identity (Nishimura et al., 2010; Susilo et al., 2010; Palermo et al., 2011). However, Palermo et al. (2011) found the difference between CP and control participants for the response to the average face: After adapting to the anti-face, the controls regarded the average face as the original (opposite of anti-face) faces, but the response of CP was chance level. Moreover, the groups appeared to differ in discrimination precision, indicating that the controls had more precise discrimination. These results suggest that CP does not make identity judgments in the same way as the controls although CP they could discriminate between identities to some extent. Face space of them were more coarse (Nishimura et al., 2010), or based on high-level object coding mechanisms that are not specific to faces (Palermo et al., 2011). Nishimura et al. (2010) examined the identity aftereffect of not only CP but also AP, who was the only one of their participants, and reported that his/her performance was dissimilar from those of control and CP participants. Particularly, he/she showed no systematic response (i.e., responses did not fit to sigmoidal curves) according to identity intensity. So far, to our knowledge, there are few studies examining difference in face adaptation between AP and CP, and no studies between the *apperceptive* and the *associative prosopagnosia*. As we have seen, face adaptation is one of the useful paradigms for examining facial representations, and more research is needed in the future on people with atypical face recognition.

## 4 Beyond visually presented face

### 4.1 Representation of non-presented face

An adaptation study revealed that non-existent visual stimuli, such as imagery, cause aftereffects as well as the face in reality. This means that there is a common neural representation that is activated by both visual and mental (imagery) faces in high-level face-perception processes. For this topic, we focus on the study of mental imagery and the ensemble of facial expressions.

We can create vivid imagery even if the visual stimuli do not exist in front of us, and it has been reported that mental imagery and perception of visual stimuli activate the same brain regions (O’Craven and Kanwisher, 2000). Consistent with these results, adaptation to mental images of faces and facial expressions induced the same pattern of aftereffects as adaptation to real visual stimuli (Ryu et al., 2008; Zamuner et al., 2017). In these studies, participants were asked to associate the models’ face identities with their names (Ryu et al., 2008) or to memorize pictures of a model expressing six basic emotions (Zamuner et al., 2017). Then, participants adapted to the realistic face or vividly visualized these faces (i.e., adapted to non-presented faces). Ryu et al. (2008) used matching anti-faces, mismatching anti-faces, and their imageries as adaptation stimuli. After adapting to matching anti-faces or vividly visualizing their faces, the intensity of the features needed to identify each person decreased compared to the control condition (in which they were not adapted to or visualized faces) and adaptation to mismatching anti-face conditions. Similarly, Zamuner et al. (2017) used a person with six basic facial expressions or imageries as adaptation stimuli, and the same facial expressions as adaptation stimuli were presented as test stimuli. After adapting to facial expressions or their images, recognition performance decreased compared with the control condition. These results indicate that the real face and imagined face shared a common representation. In addition, the results also showed that the size of the aftereffect by real faces was greater than that by imagery faces, except for surprised facial expressions. Taken together with the finding that the real face and imagery face activated the same brain region, but the real face was strongly activated (O’Craven and Kanwisher, 2000), it is suggested that the size of the aftereffect predicts the extent to which the face engages a particular neural region.

However, studies on adaptation to the sex of faces have shown inconsistent results (DeBruine et al., 2010; D’Ascenzo et al., 2014). D’Ascenzo et al. (2014) used three male and female faces as adaptation stimuli, and androgynous faces were created by morphing female and male faces as test stimuli. Participants were asked to rate masculinity or femininity by moving a slider on a scale that labeled masculinity or femininity at both ends. They reported that adapting to real faces resulted in a similar pattern of adaptation in a previous study: female judgment decreased more after adapting to female faces than to male faces. In contrast, adapting to the imagined face had the opposite pattern: female judgment increased more after adapting to female faces than male faces. They discussed the inconsistent results with Ryu et al. (2008) and suggested that different face properties in different processing evoked varied aftereffects. Based on this suggestion, it is possible that investigating the direction or strength of the aftereffect could reveal the different processing of various components of faces in imagery, and the distinction of the representation of real and imagery faces.

It is known that we can extract the average information from multiple visual stimuli automatically and rapidly, which is called the ensemble average, and it has been reported using faces (De Fockert and Wolfenstein, 2009) and facial expressions (Haberman and Whitney, 2007, 2009). This ensemble helps us understand the surrounding environment at a glance. There are two types of ensemble: the temporal statistical ensemble, in which the extracted visual stimulus is presented sequentially one by one at a time, and the spatial statistical ensemble, which involves extracting multiple visual stimuli presented simultaneously. It is important to note that the ensemble average extracted from the face groups is not necessarily the presented face. For facial expressions, adapting to sequentially or spatially presented multiple facial expressions showed the same pattern of aftereffects as when adapting to faces of the same intensity as the average of those stimuli (Ying and Xu, 2017; Minemoto et al., 2022b). Both studies have used different individuals with facial expressions as adaptation stimuli, so the average was a different individual from each model and looked more similar to morphed faces with 35 models used as test stimuli. As the size of the aftereffect was very small when the adaptation stimuli were an emotional voice or a dog’s emotional posture, but not human facial expressions (Fox and Barton, 2007), the results suggest that the ensemble average can be represented visually, and it shares a common neural representation with a real face.

To compare the adaptation to ensemble average and real faces, Ying and Xu (2017), Minemoto et al. (2022a) used a morphed averaged face with adaptation stimuli and a model with the averaged intensity of facial expressions as adaptation stimuli and showed that the sizes of adaptations to the averaged face and ensemble average were comparable. Considering that imagery adaptation leads to weaker aftereffects (Ryu et al., 2008; Zamuner et al., 2017), these results suggest that imagery and ensemble representations may differ in intensity even though they share the same neural mechanism.

### 4.2 Social message and signals of face

A face carries rich information, and they are important to building social relationships because we can recognize the personality traits, inner states, or surroundings of others based on them (Zebrowitz, 1997; Winston et al., 2002; Ekman, 2012). Thus far, we have reviewed studies on the perception of face, and finally considered the social messages or signals that were conveyed.

Previous studies have reported that adaptation also occurs with social information such as trustworthiness (Engell et al., 2010; Wincenciak et al., 2013), friendliness (Prete et al., 2018), physical strength, dominance (Witham et al., 2021), and helping judgment (Minemoto et al., 2022a). Both trustworthiness and friendliness correlate with the perception of happy and angry facial expressions: happy facial expressions are associated with trustworthiness and high friendliness, and angry facial expressions are associated with untrustworthiness and threat (Oosterhof and Todorov, 2009; Ekman, 2012). Adaptation to angry faces increases trustworthiness and greater friendliness judgments to a subsequently neutral test face rather than adaptation to happy faces (Engell et al., 2010; Prete et al., 2018). Engell et al. (2010) also showed that this trustworthiness aftereffect remained when they used different sizes of test stimuli from the adaptation stimulus (i.e., 80%), and the strength of the aftereffect was influenced by the adaptation duration. Witham et al. (2021) showed the same patterns of aftereffects for perceived physical strength and dominance. After adapting to anti-angry facial expressions, the face with average expressions of six basic emotions and a neutral emotion appeared physically stronger and more dominant, although adaptation to anti-fearful facial expressions had opposite aftereffects (i.e., physically weaker and less dominant). These results suggest that trustworthiness, friendliness, physical strength, and dominance rely on the same or partially overlapping neural mechanisms involved in the perception of facial expressions. In addition, Witham et al. (2021) reported that adaptation to anti-happy faces showed a small but similar directed aftereffect to adaptation to anti-angry faces, suggesting that the function to enhance perceptual strength could not be specific to one facial expression category.

Wincenciak et al. (2013) investigated the effect of adaptation to more and less trustworthy neutral faces on trustworthiness judgments and showed that only female participants were affected, whereas males were not. The aftereffect observed in female participants had the typical characteristic of other face aftereffects: the neutral faces were rated untrustworthy after adapting to trustworthy faces, while they were rated trustworthy after adapting to untrustworthy faces. Wincenciak et al. (2013) noted that the previous study (Stirrat and Perrett, 2010) indicated that male observers were less influenced by visual information of the face, such as width-to-height ratio, which is linked with testosterone and is predictive of aggression, than female observers. Moreover, females with more subordinate traits are more influenced by the width-to-height ratio. These results suggest that different factors and processes are involved between men and women in the perception of trustworthiness, and that the adaptation paradigm may be useful for examining the process of social messages.

Interestingly, two different facial expressions may relate to the same social message. For example, it has been reported that the perception of sad and fearful facial expressions induces prosocial behaviors in helping judgment (Marsh et al., 2005, 2007; Ekman, 2012). Minemoto et al. (2022a) reported that adapting to persons with sad facial expressions reduced their perception of the need for help (e.g., how much participants thought the person needed help) for both those with sad and fearful facial expressions, whereas adapting to those with fearful facial expressions reduced it only for those with fearful facial expressions. Considering that adaptation to facial expressions consistently reduces the perception of the same expressions (Hsu and Young, 2004; Juricevic and Webster, 2012), these results indicate that adapting to sad facial expressions influences not only facial expression perception but also social signal processing. Given that adaptation and aftereffects occur automatically, people automatically perceive the need for help when they see sad facial expressions. By contrast, fearful facial expressions do not have this function, although both expressions give observers the impression of the need for help.

Currently, few adaptation studies have focused on social messages. However, as presented here, the adaptation paradigm can be a useful tool for investigating what signals we automatically process when we see faces and what is based on our social judgments.

## 5 Discussion: The future of the adaptation paradigm

Face identity and facial expressions are important social cues for communicating and establishing social relationships with others, and the adaptation paradigm is a good procedure to examine their process and representation. In this study, we reviewed behavioral studies on the aftereffects of face identity and facial expressions from the basic characteristics that have been observed in various studies to the still discussing topics.

Early studies using adaptation, with typical adult participants using realistic face stimuli, have provided widespread support for the idea that norm-based coding is used for face recognition processes (Leopold et al., 2001; Juricevic and Webster, 2012). Subsequent studies have provided a distinct or overlapping relationship between identity and facial expressions, which has already been suggested (Bruce and Young, 1986; Haxby et al., 2000; Calder and Young, 2005), by using the validity of facial models (Fox and Barton, 2007; Fox et al., 2008).

Studies on participants with immature or impaired face recognition (child, ASD, and prosopagnosia) reported that aftereffects were observed, suggesting that processes other than norm-based coding may be responsible for their atypical face processing (Burton et al., 2013; Jeffery et al., 2013). However, there are some problems with this topic. First, few studies have explored the aftereffects of facial expression in children. As Vida and Mondloch (2009) suggested, different developments were observed in different category boundaries, and other types of category boundaries of facial expressions need to be examined to understand the development of facial expressions. Second, ASD studies have proposed that ASD has an atypical relationship between the categories of facial expressions compared to TD, and the findings of the prosopagnosia study suggest that CP has an atypical face space. Further research is needed to explore the representation of facial expressions of people with deficits in facial recognition.

There has been a growing body of aftereffect research on information related to non-presented faces, such as imagery faces (Ryu et al., 2008; Zamuner et al., 2017), face ensemble averages (Ying and Xu, 2017; Minemoto et al., 2022b), and the social messages or perceived personality traits of facial expressions (Engell et al., 2010; Wincenciak et al., 2013; Prete et al., 2018; Witham et al., 2021; Minemoto et al., 2022a). The results indicated that non-presented faces also induced the aftereffect, and they showed basically the same pattern as a realistic face. In addition, social messages and perceived personality traits affected adaptation to facial expressions. This topic is one of the future directions for adaptation studies: investigating social messages or perceived personality through the face (hereinafter referred to as the “social roles of the face”). The social role of the face is essential for building relationships with others. Despite this, previous adaptation studies have mainly shed light on the characteristics and representations of faces and have not yet examined much of the representations and cognitive functions of the impressions we receive from faces. To examine its cognitive processes, an adaptation paradigm can reveal the function of various types of facial information (e.g., anger is related to trustworthiness). As there are so many different types of social roles of the face, related studies are still limited. As reported in Section 4.2, we would say that this topic has a wide range of unexamined aspects. If the recognition of the social roles of faces is not dependent on the perception of facial expressions, then there may be a group that is accurate in the perception of facial expressions but is unable to recognize the social roles of faces and struggle with it. Therefore, this topic will need to continue to be considered in the future.

Finally, we recapitulated the advantage of the method of adaptation compared with the direct response to stimuli. The adaptation paradigm can eliminate unexpected factors because the same test stimuli and tasks were used both before and after the adaptation phases. Specifically, task demands are often inferred when participants respond to stimuli, and this is more likely to occur in face research because faces are strongly social. The adaptation paradigm may avoid this serious issue by examining the shifted responses before and after adaptation; that is, we could investigate the difference regardless of the participants’ attitude. This advantage is particularly useful when considering social messages in which the task demand is easily guessed.

Recently, we have been able to consider that face adaptation studies have approximately reached the stage of revealing the basic features of facial representations. However, as discussed in this review, the potential for new applications of the face aftereffect remains open.

## Author contributions

KM and YU conceptualized the review. KM was primarily responsible for the article research and drafted the original manuscript. YU revised and supervised the manuscript. Both authors approved the final version of the manuscript.

## Footnotes

1 The central point of the face space is referred as “average,” “norm,” and “prototype.” Moreover, they are often interchangeably used (e.g., Rhodes et al., 1987; Valentine, 1991). Although it is better to use them differently based on the roles or functions of them, the differences among them seem not to be clearly defined yet. In this study, the following remarks are made to ensure that these terms are commonly used in studies of facial identity and facial expression. First, in the facial expression studies, the faces of neutral category are often used as “norm.” “Average” is not appropriate in this field because the neutral faces are not made from other facial expressions. Second, “prototype” sometimes refers to typical facial expressions (e.g., Ekman et al., 1983). Therefore, the term “norm” generally refers to the center of the face space, whether it is used for identity or facial expression. In this study, “norm” is used to refer to this, and “average” is defined as the average over multiple faces using morphing techniques. “Prototype” does not used because it could be mistaken for its opposite meaning in facial expression studies.

2 Since identity A and anti-A are in the opposite direction across the average in the face space, the results after adapting to identity A and anti-A are also the opposite. For example, adapting to identity A makes the average look less like A though adapting to anti-A makes the average look more like A.

3 Matching anti-face and mismatching anti-face were referred to as opposite face and non-opposite face, respectively, by Rhodes and Jeffery (2006), but we use the term “anti” in this paper for consistency.
